# Resilience through risk management: cooperative insurance in small-holder aquaculture systems

**DOI:** 10.1016/j.heliyon.2018.e00799

**Published:** 2018-09-26

**Authors:** James R. Watson, Fredrik Armerin, Dane H. Klinger, Ben Belton

**Affiliations:** a Oregon State University, College of Earth, Ocean and Atmospheric Sciences, Corvallis, USA; b Department of Real Estate and Construction Management, KTH Royal Institute of Technology, Stockholm, Sweden; c Center on Food Security and the Environment, Stanford University, Stanford, CA, USA; d Department of Agricultural, Food, and Resource Economics, Michigan State University, East Lansing, USA

**Keywords:** Environmental science, Economics, Agriculture

## Abstract

Aquaculture is a booming industry. It currently supplies almost half of all fish and shellfish eaten today, and it continues to grow faster than any other food production sector. But it is immature relative to terrestrial crop and livestock sectors, and as a consequence it lags behind in terms of the use of aquaculture specific financial risk management tools. In particular, the use of insurance instruments to manage weather related losses is little used. In the aquaculture industry there is a need for new insurance products that achieve both financial gains, in terms of reduced production and revenue risk, and environmental wins, in terms of incentivizing improved management practices. Here, we have developed a cooperative form of indemnity insurance for application to small-holder aquaculture communities in developing nations. We use and advance the theory of risk pools, applying it to an aquaculture community in Myanmar, using empirical data recently collected from a comprehensive farm survey. These data were used to parameterize numerical simulations of this aquaculture system with and without a risk pool. Results highlight the benefits and costs of a risk pool, for various combinations of key parameters. This information reveals a path forward for creating new risk management products for aquaculturalists around the world.

## 1 Introduction

Aquaculture is one of the most diverse and fastest growing food production sectors on the planet (Pulvenis, 2016). It is also a highly heterogeneous sector, with farm enterprises ranging from low-input, land-based ponds maintained by individual subsistence farmers to high-input coastal cages owned by transnational corporations (Little et al., 2016; Ottinger et al., 2016). Economic risks are ubiquitous across the sector: aquaculture is inherently risky and often has higher variability in both yields and revenues relative to other food production systems (Kam and Leung, 2008; Engle, 2010; Flaten et al., 2011). This is due in part to the growth of aquatic organisms (which are mostly ectothermic) being highly sensitive to changes in environmental conditions and on the immaturity of the technology used by the majority of the aquaculture industry, relative to many agricultural and livestock producers (Tidwell and Allan, 2012; Kumar and Engle, 2016). As a consequence of the inherent variability in fish-farm revenue, and the lack of demand and availability of risk management products, currently only a small fraction of the aquaculture industry is insured for losses (Secretan et al., 2007; Beach and Viator, 2008). This is in stark contrast to insurance in agriculture, where economic risk-management tools like insurance are far more wide-spread (Moschini and Hennessy, 2001).

The lack of production and/or revenue insurance in aquaculture has helped create a number of economic and environmental problems (Van Anrooy, 2006; Secretan et al., 2007). While the aquaculture industry as a whole is growing rapidly, growth in some countries is slow, and in a number of systems there is a high turnover of aquaculture producers, as many entities exit the industry after initial failures (Nash, 2011). Further, to mitigate the inherent risks associated with fish farming, aquaculturists will often employ inefficient management practices, such as the overuse or prophylactic use of therapeutants and antimicrobials (Rico et al., 2012; Cabello et al., 2013). This can help sustain large and consistent yields in the near-term, but it often comes with a longer-term environmental cost, which can ultimately lead to catastrophes that impact not only individual growers, but also their neighbors as these negative impacts spill over (Asche et al., 2009).

Financial tools like insurance can help incentivize food producers, including aquaculturists, to adopt best management practices that reduce local and regional environmental impacts and hence diminish the risk of regional catastrophes (Coble et al., 2003; Tucker et al., 2009). For example, when insurance policies for intensive shrimp pond operations require that farmers reduce the rate of water exchanged with surrounding water bodies, the amount of pollution from farm effluent is decreased, resulting in reduced environmental harm and reduced risk of disease exposure for other farms that utilize the same water body (Menasveta, 2002; Secretan et al., 2007). Policies that require established best management practices (e.g. specific stocking densities) to avoid undue stress often result in decreased disease prevalence and reduced need for excessive antimicrobial usage (Henriksson et al., 2017). However, these gains can only be realized if and when insurance products are designed appropriately for aquaculture production systems.

Here, we have developed a cooperative form of aquaculture indemnity insurance aimed at harnessing the often strong social-capital of small-holder food production systems (Adger, 2000). The approach hinges on the concept of a cooperatively managed mutual fund, where aquaculturists self-organize into a cooperative that self-insures using this fund. Members of the cooperative also monitor and verify losses. This approach to insurance is not new and it has been applied in numerous cases in agriculture (Cone and Myhre, 2000) and in a limited number of cases in aquaculture (Nissapa et al., 2016; Xinhua et al., 2017). Indeed the cooperative model of insurance is at the heart of Protection and Indemnity Clubs (Bennett, 2001), which have been active in the maritime transportation industry for over a century, as well as in new insurance companies like Lemonade (https://www.lemonade.com) and Friendsurance (https://www.friendsurance.com/) which provide home insurance to the public, and most recently the new decentralized approaches to insurance based on cryptocurrencies (e.g. https://etherisc.com/). We generalize this approach for application to the aquaculture industry, where fish farmers who wish to be compensated for downside risk do so by forming an insurance risk pool.

## 2 Methods

We have designed a cooperative form of indemnity insurance, and explored its utility using numerical simulations parameterized with empirical economic data collected from a fish farming community in Myanmar, who are adversely affected by heavy rainfall and subsequent flooding. The methods below first introduce the social, biological, and physical characteristics of this case-study system. Then, our insurance theory is described, which we note is general to any group of food-producers aiming to self-insure against losses through a risk pool. Last, we describe numerous simulation experiments used to explore the benefits and costs associated with an aquaculture risk pool in Myanmar.

### 2.1 Myanmar aquaculture as a case-study

The aquaculture sector in Myanmar serves as a valuable example of a rapidly expanding food-production sector providing an important source of food and income locally but that has little to no access to financial risk-management tools (Van Anrooy, 2006). Aquaculture production in Myanmar, which is almost exclusively for finfish, has grown rapidly over the last two decades and plays an increasingly important role in national fish supply (Belton et al., 2015). The sector's technical and economic characteristics have been studied in a recent survey – the Myanmar Aquaculture–Agriculture Survey (MAAS). Here, we only provide a brief description of the main methodological steps in MAAS, as there are detailed reports elsewhere (Belton et al., 2017).

**Figure 1:**
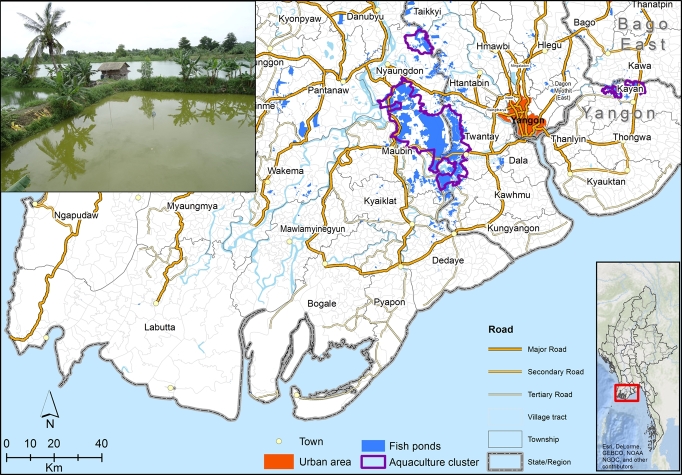
Map of the aquaculture study region near the capital Yangon in Myanmar (larger-scale map inset on the right). Inset on bottom left – a photo of a typical aquaculture pond in this region.

MAAS was implemented in May 2016 and data were collected from a total of 1102 rural households for the preceding year (2015), including crop farmers, fish farmers, and the landless, located in 40 village tracts in four townships (Twantay, Maubin, Nyaungdon, Kayan) in the Ayeyarwady and Yangon regions. All the village tracts surveyed lie in a zone within a radius of 60 km from Myanmar's largest city and main commercial center, Yangon. The households surveyed represent a total population of about 37,000 households. A subset of 242 fish farming households (151 growout farms and 73 nurseries) were interviewed in 25 village tracts (the “aquaculture cluster” village tracts identified in Figure 1), representing a total of 2450 fish farming households. The surveyed fish-farms represent 57% of the total area of inland fish ponds in Myanmar (see Table 1). Farms surveyed were selected to represent the entire population of fish farming households resident in the 25 village tracts. Given that 90% of Myanmar's inland fish ponds are located in the Ayeyarwady and Yangon regions (Belton et al., 2017), the sample can be considered to represent approximately half the area used for freshwater aquaculture in Myanmar.

**Table 1:** Summary of Myanmar fish-farm empirical data.

	min	mean	std	max
Farm area (ha)	0.24	8.84	16	162.00
Production per farm (tons)	0	33.67	41.25	203.20
Gross revenue per farm ($)	500	49900	73700	417000
Operating cost per farm ($)	304.17	33192.00	39400	182000
Flood Impact (%)	10	40	24.65	100

**Figure 2:**
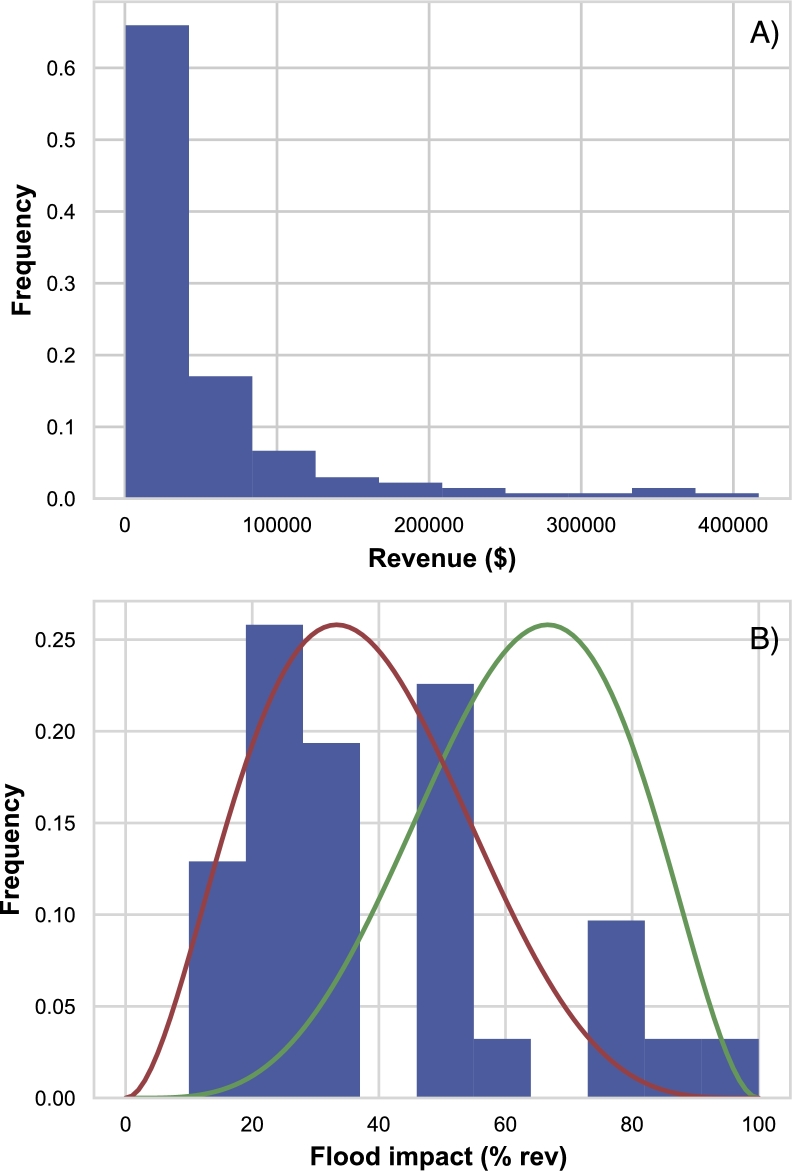
A) The empirical revenue distribution for the Myanmar fish farm system, including data from 151 farms, for 2015. B) The empirical distribution of flood impacts (as a fraction of revenue lost), with two modeled flood impact distributions. Flood impacts are modeled using a beta distribution, where parameters can be chosen to simulate environmental conditions that lead to frequent weak (red curve) and strong (green curve) flood impacts.

Two types of fish farms were surveyed: 1) specialized nurseries growing juvenile fish (“fingerlings”) for sale to growout farms (41% of fish-farms); and 2) “growout” farms producing food fish for the market (59% of fish-farms). All subsequent analyses pertain to the growout farms, and the damage that flooding has on their production of fish, and hence revenue. Food-production by these aquaculturalists can be strongly affected by floods, which cause damage to the farming infrastructure and can literally flood the ponds, causing fish to escape. Data from the interviews with the aquaculturalists identify that floods can reduce annual revenue by up to 80% (see Figure 2), and in 2015 (the year before the interviews) approximately 40% of households reported some losses due to flooding, and 15% of households estimated that their losses amounted to more than 30% of their expected production.

### 2.2 Modeling an aquaculture risk pool

#### 2.2.1 Basic insurance mathematics

In order to model the impact of an aquaculture risk pool in Myanmar, we must pose the problem in terms of standard insurance mathematics and notation. So, consider an insured person (or firm) who owns a fish farm, who has wealth *w*, and a utility function *u* which is continuous, non-decreasing and concave. Further, potential losses by flooding is given by the random variable *L*. In the case that this loss is fully insured, then any premium Π, paid by the insured and which satisfies the inequality

(1) E[u(w−L)]≤E[u(w−Π)],

is acceptable to the insured fish farm (Kaas et al., 2008). In addition, the premium $\Pi^{+}$ satisfying the equality

(2) $$E[u(w-L)]=E[u(w-\Pi^{+})]$$

is called the zero utility premium (Kaas et al., 2008), which is important for if *u* is continuous and non-decreasing we have

(3) $$E[u(w-L)]\leq E[u(w-\Pi )]\text{ if and only if }\Pi \leq \Pi^{+}.$$

Hence, $\Pi^{+}$ defines the maximum premium the insured is prepared to pay. If, more generally, the loss is only compensated by the amount *X*, where 0≤X≤L, then the inequality above is replaced by

(4) E[u(w−L)]≤E[u(w−L+X−Π)].

The zero utility premium in this case is determined by

(5) $$E[u(w-L)]=E[u(w-L+X-\Pi^{+})],$$

and again we have the interpretation of $\Pi^{+}$ as being the maximum premium an insured fish farmer is prepared to pay:

(6) $$E[u(w-L)]\leq E[u(w-L+X-\Pi )]\text{ if and only if }\Pi \leq \Pi^{+}.$$

In the case when the insured is risk-neutral, their concern is only the expected value of the payoff, and this can be represented by the utility function

(7) u(x)=x.

In this case we have

(8) Π≤E[X]

and

(9) $$\Pi^{+}=E[X]$$

respectively. Using $\Pi^{+}$ as the premium describes the situation where the fish farmer is indifferent about being insured and not being insured. In the cooperative risk pool model, where the insured put the premiums into a mutual fund, the zero utility premium is an equilibrium in which the total amount put into the mutual fund is maximized. For this reason the zero utility principle gives us a suitable principle for setting the premiums in risk pools.

#### 2.2.2 The risk pool model

With the basic insurance notation in-hand, we now consider *n* fish farms, each of whom i=1,2,…,n pay a premium $\Pi_{i}$ into a risk pool mutual fund (see Table 2 for an overview of the main variables and parameters in the insurance model). We make the common assumption that the fish-farmers are risk-averse, but see Maart-Noelck and Musshoff (2014) who report that some farmers may be risk-seeking. The normal next step would be to then assign to each fish-farmer a utility function reflecting this risk preference. However, given that in general it is extremely difficulty of quantify a person's utility function, we instead use the following pragmatic approach and set the premium according to

(10) $$\Pi_{i}=E[X_{i}].$$

**Table 2:** Parameters of the cooperative insurance risk pool model.

Parameter	Symbol
Farmer information	
The number of farmers	*n*
Revenue	*R*_*i*_
Loss due to floods	*L*_*i*_

Weather information	
Probability of flooding	*p*
Flooding indicator (in {0,1})	*I*_*i*_
Impact of flooding (fraction of revenue)	*ξ*_*i*_

Risk pool parameters	
Premium	Π_*i*_
Coverage (fraction of loss)	*δ*
Payout	*X*_*i*_
Limited payout	${\overset{ˆ}{X}}_{i}$
Risk pool mutual fund value	*M*
Limited risk pool mutual fund value	$\overset{ˆ}{M}$

We further assume that the pay-out from the mutual fund in case of a loss $L_{i}$ is given by $X_{i}=\delta L_{i}$ for some δ∈(0,1) common to all farms. The loss farm *i* faces is modeled as

(11) $$L_{i}=R_{i}I_{i}\xi_{i},$$

where

(12) $$P(I_{i}=0)=1-p\text{ and }P(I_{i}=1)=p$$

for some common probability p∈(0,1) and $\xi_{i}$ is a random variable with support on [0,1]. All $I_{i}$ and $\xi_{i}$ are assumed to be independent of each other. The interpretation is that with probability *p*, fish farm *i* is exposed to a flood (represented by $I_{i}=1$), and then given that there is a flood, the fraction $\xi_{i}$ of their revenue $R_{i}$ is lost. With this specification we have

(13) $$\Pi_{i}=E[\delta L_{i}]=\delta E[R_{i}I_{i}\xi_{i}]=\delta R_{i}pE[\xi_{i}].$$

The premiums from all the farms are put into the mutual fund at the beginning of the year. After one year, the losses are known and the value of the fund after payouts is

$$\begin{array}{ccc} M & = & M_{0}+\sum_{i=1}^{n}\Pi_{i}-\sum_{i=1}^{n}X_{i}\\ & = & M_{0}+\sum_{i=1}^{n}\delta R_{i}pE[\xi_{i}]-\sum_{i=1}^{n}\delta R_{i}I_{i}\xi_{i}\\ & = & M_{0}+\sum_{i=1}^{n}\delta R_{i}(pE[\xi_{i}]-I_{i}\xi_{i}), \end{array}$$

where $M_{0}\geq 0$ is the initial capital of the fund. To include dynamics in the model, a time index is required. To do so it is first assumed that farm revenue (in the absence of any loss incurred by a flood), while heterogeneous across farms, is constant over time. This is obviously a strong assumption, as farm revenue will vary from year to year. However, this choice reflects the Myanmar data, which only describes revenue for one year (2016). This simplifying assumption leads to the relation:

(14) $$M_{t}=M_{t-1}+\sum_{i=1}^{n}\delta R_{i}(pE[\xi_{i,t}]-I_{i,t}\xi_{i,t}),$$

where $M_{t}$ is the value of the mutual fund at time *t* after payments have been made. Note that each farmer pays their premium at the beginning of each year, so there is always an inflow of money each year. We assume that there is independence over time, i.e. that every $I_{i,t}$ and $\xi_{i,t}$ are independent.

An important question is what happens if there is not enough money in the mutual fund to cover all the claims. In the risk pool scheme, payouts are effectively stopped if there is not enough money in the mutual fund. In this case, the expected net profit of the risk pool insurance scheme is negative. Hence, it will not be acceptable for a risk-neutral fish farmer to enter into the risk pool. Indeed, even a fish farmer that is risk-averse but close to risk-neutral, might not choose to enter the risk pool. But, in this model we assume that all fish farmers have a sufficient level of risk-aversion that makes this insurance scheme attractive.

The likelihood of the mutual fund running out of money is referred to as the probability of ruin, i.e. the probability that there is not enough money to cover the claims, and it is an important and well studied entity in insurance mathematics (e.g. Browne, 1995). To calculate (or at least estimate) the ruin probability in our risk pool model we used numerical simulations, although there are also analytical methods (Kaas et al., 2008). Because the insured fish-farmers essentially own their mutual fund, ruin can be avoided, and in our model we implemented one possible solution: if the total claims exceeds what is in the mutual fund, then each fish farmer who is making a claim is paid in proportion to their loss and in such a way that the whole fund is used, but not more. With this modified method, the payout from the mutual fund after one year to fish-farmer *i* is given by

(15) $${\overset{ˆ}{X}}_{i}=\min\left(X_{i},\frac{X_{i}}{\sum_{j=1}^{n}X_{i}}M\right),$$

where *M* is the value of the mutual fund **before** the payments are made, and the value of the fund **after** the payments are made is $\overset{ˆ}{M}=\max\left(M-\sum_{i=1}^{n}X_{i}\right)$. This is then iterated through the life time of the risk pool. If at any time all $X_{i}$s are equal to zero then ${\overset{ˆ}{X}}_{i}=0$. Since the average profit and loss of the mutual fund when there is no downside limit over a year is equal to zero, in this sense the net principle is the “fair market premium” (Mikosch, 2009). In general, the fair market premium is considered too small to be charged by an insurer since the insurer will be ruined with probability one, and it is known to be of “purely theoretical value” (Mikosch, 2009). However, since a risk pool is a mutual insurance construction, i.e. the insured are also the insurers, and in our suggested implementation of the risk pools, if there is no money left in the fund then the payments are cancelled (so in practice there will be no ruin), we believe that this assumption is a reasonable starting point for modeling premiums in a risk pool. Indeed, the fact that the risk pool mutual fund can run out of money is not a problem per se. Each of the fish farmers will be repaid using all available funds, and in proportion to their respective losses.

### 2.3 Simulation experiments

The risk pool theory presented above was used to develop several simulation experiments, from which we assessed the potential benefits and costs of a risk pool in the Myanmar aquaculture system. In these simulations, the dynamics of a region-wide risk pool, where all 130 Myanmar fish-farms are members, is integrated over a 25 year period at yearly intervals. This integration period reflects the typical life-time of a fish-farm in Myanmar (as per communications with the fish-farmers during the MAAS survey). Following the risk pool theory, we assumed that the revenue before losses is constant through time, but heterogeneous across farms, with values taken directly from the MAAS data. We also assumed that the premiums are set using Equation (13), that for each simulation the model parameters are fixed throughout the integration period, and that there is no discounting/accumulation, i.e. interest rates are equal to zero. Last, for every simulation experiment we performed numerous realizations of each parameter set, because flooding and flood impacts were modeled probabilistically, which were then summarized using ensemble statistics.

To develop the simulation experiments, we first had to specify probabilistic models for flooding and flood impacts. Flooding was modeled simply as a Bernoulli random variable, with the likelihood of flooding a free-parameter that we explored. Flood impacts (i.e. the fraction of revenue lost per fish-farm, given a flood) was modeled using a beta-distribution, which is a flexible distribution with two parameters: *α* and *β* (the same for all farms). A beta-distribution was fit to the empirical data (Figure 2B, red line), leading to values of α=2.5 and β=5.1. These parameter values create a flood impact distribution with a mode at low values (mean flood impact =32%) and a tail to the right, allowing for potentially very high impact floods. To explore scenarios where high impact floods are more frequent, as they may be in the future under climate change, we flipped these parameters values (α=5.1 and β=2.5); this flood impact distribution has a mode to higher impact values (Figure 2B, green line, mean flood impact =67%). The two beta-distribution parameters sets describe “weak” and “strong” flooding scenarios respectively.

With these specifications, we explored simulation outcomes over a range of values for the probability of flooding *p*, and the fraction of revenue covered *δ* (common to all farms). The first simulations were designed to explore the probability of ruin as a function of different approaches to seeding the risk pool's starting capital. Next, following the theory above for the case where a risk pool's mutual fund is limited to values greater than or equal to zero, payouts can be less than claims, and we assessed the impact of a limited mutual fund using the ratio of payouts to claims in the *p* and *δ* parameter space. Last, we performed simulations to quantify the impact of a risk pool on individual farm cash flows under the “weak” and “strong” flooding scenarios. To measure this impact, we calculated the ratio of the 5th percentile of the income distribution with and without a risk pool. Essentially, this metric measures how much the risk pool brings up the downside of the income distribution. We calculated these values in the *p* and *δ* parameter space, and compared them to changes in the median income with and without a risk pool, and also the premium that must be paid in order for the risk pool to be operational.

## 3 Results

**Figure 3:**
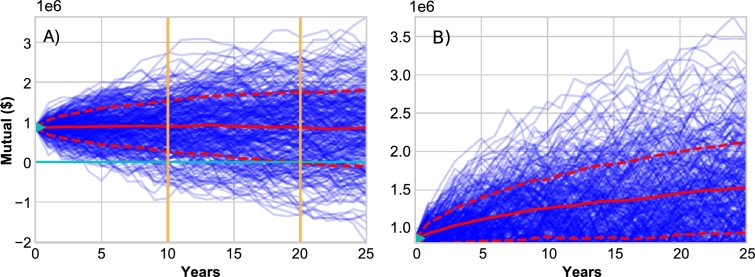
A) Simulated mutual fund trajectories in blue (in United States Dollars $), for a case where the risk pool's mutual fund is allowed to be negative. These trajectories highlight the probability of ruin, which is a function of both the starting capital, identified by the green triangle at year zero, and the integration period of the simulation (25 years), for example as identified by the two vertical gold lines. The mean, and 25th and 75th percentile trajectories are identified by the solid and dashed red lines respectively. B) Mutual fund with negative values are not realistic in the Myanmar aquaculture system, and hence we developed simulations where payouts follow Equation (15), where payouts are limited to what is in the mutual, and as a consequence risk pool mutuals remain greater than or equal to zero.make red lines thicker.

Initial simulation experiments were performed to identify important qualitative features of the risk pool model. In particular, we analyzed the evolution of risk pool mutual funds though time. For example, see Figure 3), where the blue trajectories identify mutual fund trajectories for different realizations of the same parameter sets. In these simulations, the mutual funds often go negative, as does the lower dashed red line, which identifies the lower level of the confidence band over realizations. A mutual fund with negative value is not an impossibility, as it represents a scenario where the fish-farmers have access to credit. However, is it highly unrealistic, and as a consequence we updated the implementation of the risk pool simulations to include constraints on the possible payouts following Equation (15), i.e. if the total claims exceeds the amount in the mutual fund, then all the remaining capital in the mutual fund is used, but not more, and payouts are provided to individual fish farms in proportion to their claim's size. This constraint limits the mutual fund to positive values only (see Figure 3B and notice that all the trajectories and ensemble statistics are now strictly positive). One note about Figure 3: recall that the value of the mutual fund is increased at the beginning of each year by the sum of the premiums, so the value of the fund will be reset to at least the sum of the premiums at the beginning of each year.

Ruin probabilities are largely controlled by the initial capital in the mutual fund, and the life-time of the risk pool (i.e. the integration period of the simulation). We explored two ways in which initial capital is provided to the mutual fund: (1) when the initial capital is set as the sum of all premiums, as calculated following Eq. (13), which is proportional to the probability of flooding, and (2) when the initial capital is simply a fixed amount, for example the sum of a given fraction of each fish-farm's expected annual revenue. For the latter, expected annual net revenues were derived from the empirical MAAS data, and the fraction of this value committed to initialize the mutual fund was set to 10% (see Figure 3 green triangle at year 0). The following results were qualitatively consistent, regardless of the choice of this value.

**Figure 4:**
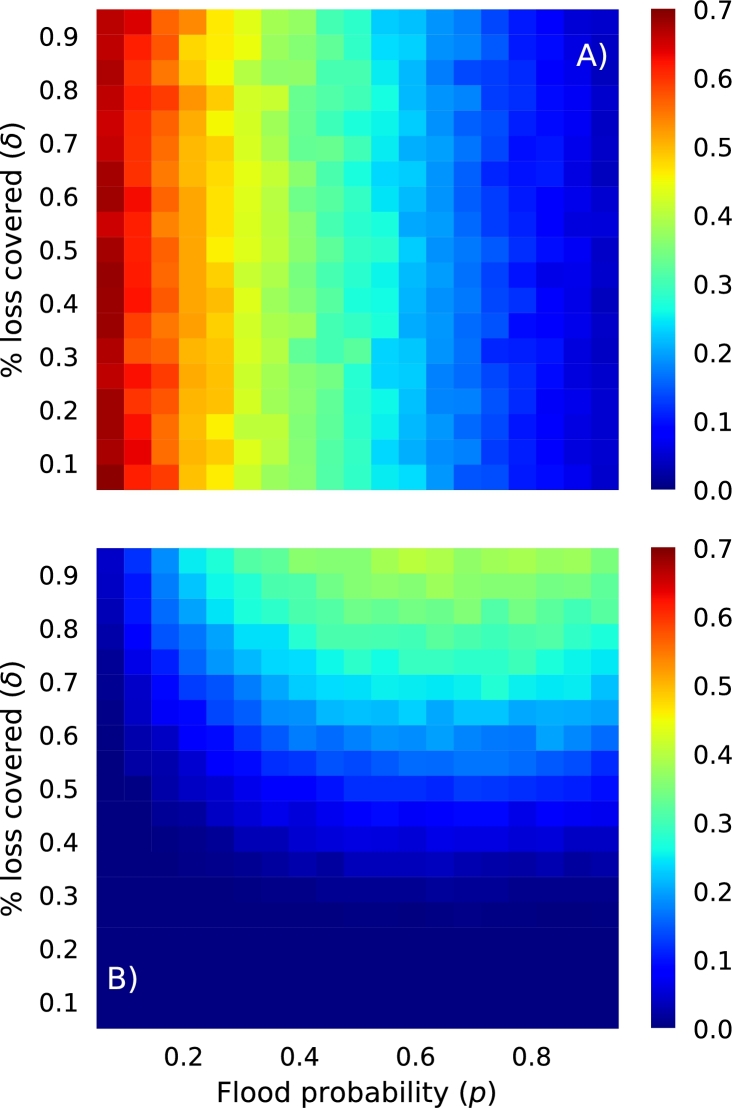
Ruin probabilities for various combinations of flood probabilities (*p*) and the fraction of loss covered (*δ*), for two different starting capital options: A) where the initial capital is the sum of premiums. In this case the starting capital is proportional to the probability of flooding *p*. B) Where the initial capital is a set fixed amount, in this case the sum of 10% of each fish farms expected revenue. In this case the starting capital is not proportional to the probability of flooding.

In the case where the starting capital is the sum of premiums, and hence proportional to the flood probability *p*, the mutual fund ruin probability is essentially invariant with the fraction of loss covered *δ*, and has a negative relationship with the probability of flooding (Figure 4A: see landscape changing from red to blue as the flood probability *p* increases). This is a non-intuitive result, as one would expect the probability of ruin to increase with the probability of flooding. However, from Equation (13) we can see that premiums scale with the flood probability *p*, and hence as *p* increases so do premiums, and so does the starting capital in the mutual fund. In the case where the initial capital is a fixed amount, ruin probabilities have a very different relationship with the flood probability *p* and the fraction of loss covered *δ* (see Figure 4B). Now, the mutual fund ruin probability has a positive relationship with both factors, with highest ruin probabilities (around 35%) occurring at high *p* and *δ* values.

**Figure 5:**
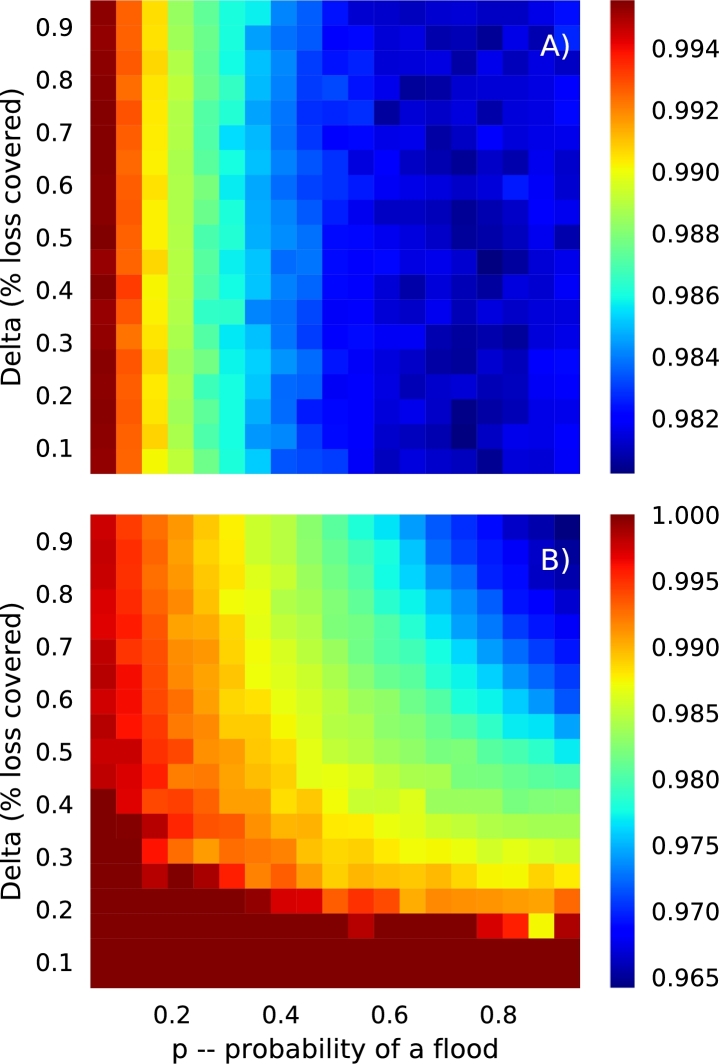
In the case where mutual funds are limited to values greater than or equal to zero, payouts can be less than what is claimed. Here, we show the ratio of payouts and claims for a range of flood probabilities *p* and values of the fraction of loss covered *δ*. Values that are blue and green identify situations when the risk pool cannot provide all that a farm might claim. These results are shown for the case when the initial capital in the mutual fund is the sum of all premiums and proportional to the probability of flooding (A) and when it is a fixed amount and not proportional to the probability of flooding (B).

In the case where the risk pool's mutual fund is limited to positive values, which is when payouts may be less than what is claimed, another important metric than can be calculated is the ratio of payouts to claims, averaged over farms. This metric identifies situations when the risk pool mutual fund provides payouts that are less than what is claimed (see Figure 5). In the case of the initial capital being the sum of premiums, the pattern in this metric mirrors that of ruin probabilities in the unlimited mutual simulations, that is the difference in payouts and claims increases with flood probability, but is invariant with the fraction of loss covered *δ*. In the case of fixed initial capital, the difference between payouts and claims is highest (i.e. near unity) at low values of *δ* and all values of *p*, but decreases as both factors increase. Interestingly, the fraction of a claim not provided is at most only around 3–4%, which may be an acceptable amount for the Myanmar fish-farmers.

**Figure 6:**
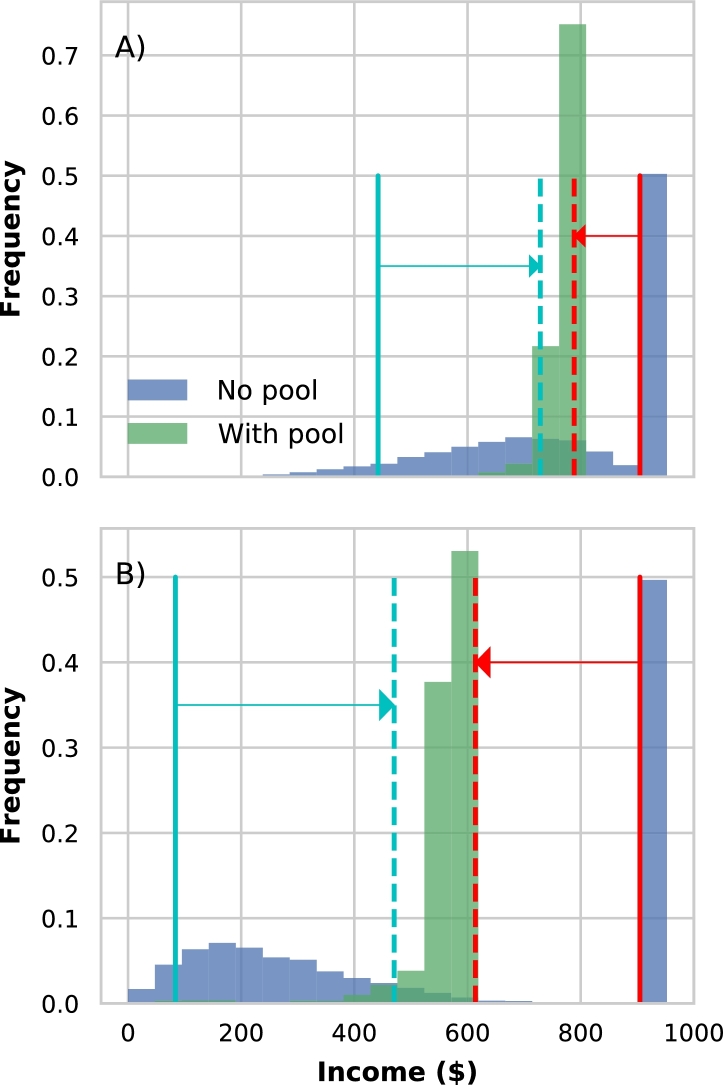
Income distributions for an individual fish farm with (green) and without (blue) a risk pool, for environments with weak (A) and strong (B) flood impacts respectively. Distributions are produced from repeated simulations with a 25 year integration period, and where the mutual fund is bound to positive values. It is evident that in all cases, the fish farm's income is characterized by a mixed distribution, with a delta peak identifying the no-flood income to the right, and a loss-distribution to the left created by years where floods were experienced. Participating in the risk pool shifts the loss-distribution to higher income values, as highlighted by the shift in the lower 5th percentile (solid and dashed vertical turquoise lines). However, this comes at a cost, reflected in the change in the no-flood income peak, which is moved to lower values due to the payment of premiums (compare the solid and dashed vertical red lines).

Every risk pool simulation creates an income distribution over time for each farm. Income is defined as the net revenue minus losses due to flooding. In the case of where a risk pool is formed, premiums are an additional loss term when calculating income. In Figure 6A we show the income distributions for an individual farm that experienced weak floods for the no-pool and with-pool simulations (i.e. the parameters of the beta distribution relate to the red line in Figure 2B). Several features become evident. The first is that in both cases income is comprised of a mixture of distributions: a delta distribution describing income when there is no flood, and a broader loss-distribution describing income when floods are experienced. The with-pool delta distribution is to the left of the no-pool delta distribution, and this difference identifies the additional cost of the premium when joining a risk pool. We see that the no-pool loss distribution extends towards to the zero-income point, while when there is a risk pool the loss distribution is constrained to larger positive values (compare the width and location of the blue and green distributions in Figure 6A). This identifies the positive impact of the risk-pool on the farmer's down-side risk. These results are entirely expected following the mathematics described previously.

In Figure 6B we show these same distributions but for a scenario where flood impacts are high (i.e. when the flood impact beta distribution relates to the green line in Figure 2B). In this case, the difference between the no-flood income delta distributions are accentuated. This is because the premiums are higher in this strong flood scenario (i.e. premiums are proportional to the expected flood impact). However, the differences in the loss-distributions are also greater, with the loss distribution for the case where there is a risk pool (Figure 6B, green) now far further to the up-side of the no-pool loss distribution (Figure 6B, blue), which is now much closer to the zero-income point.

**Figure 7:**
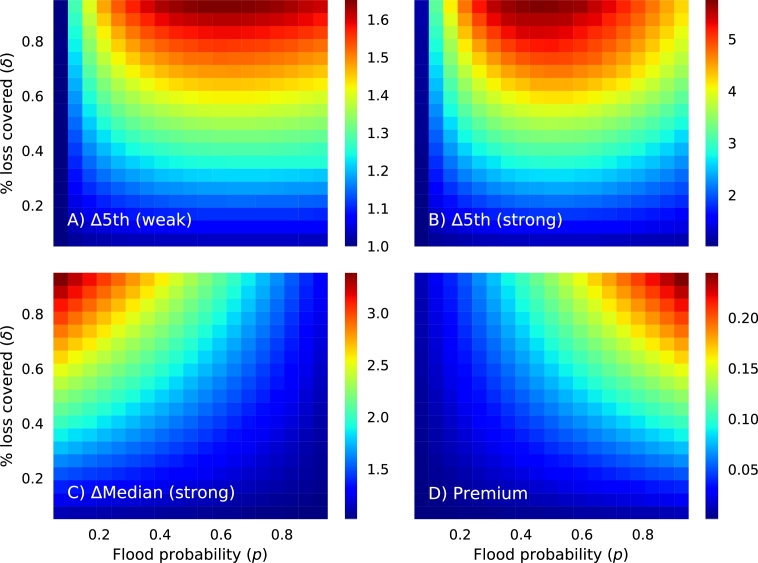
The ratio of the lower 5th percentile of the with-pool and no-pool loss distributions averaged across farms, for a range of flood probabilities *p* and values of the fraction of loss covered *δ*, for weak (A) and strong (B) floods. C) The ratio of the median of the loss-distributions for with-pool and no-pool cases, for strong flood impacts (results from weak flood impacts are not shown but show exactly the same qualitative patterns, but with maximal values around 1.5). D) The premium paid into the risk-pool mutual fund, posed as a fraction of a farm's annual revenue, for the strong flood impact scenario.

These differences between with-pool and no-pool cases is the same across all fish-farms, and taking advantage of this uniform impact of a risk pool, we measured its net benefit on the whole Myanmar fish-farming community as the ratio of the 5th percentile in the loss-distribution between with-pool and no-pool cases, averaged over all farms. We term this dimensionless ratio Δ5, and we quantified it for a range of flooding probabilities *p* and values of the fraction of loss covered *δ*, also for weak and strong flood impact scenarios (see Figure 7A and B). Intriguingly we find a non-monotonic relationship: in the case of weak floods, there is a positive relationship between Δ5 and the probability of flooding *p* and the fraction of loss covered *δ*, but with a peak at intermediate flood probabilities (i.e. in Figure 7A, the brightest reds are at *p* values around 0.7). This peak in Δ5 is accentuated in the strong flood case (Figure 7B), where maximal values are now shifted to the left, occurring when flood probabilities are around 0.5. Furthermore, maximal values of Δ5 are around 1.5–1.6 in the weak flood impact scenario, and 5–5.5 in the strong flood impact scenario. In other words, the risk pool fish-farmers are exposed to much less risk in their income when there is a flood.

Two other important metrics are the ratio of the median of the loss-income distribution, which we call Δ50 (Figure 7C), between cases with and without a risk pool, and the premium paid posed as a fraction of expected revenue (Figure 7D). Δ5 values have a positive relationship with the fraction of loss covered *δ*, and a negative relationship with the probability of flooding *p*. This identifies that most of the positive impact of a risk pool, in terms of Δ50, occurs when the probability of flooding is low and when the fraction of income covered is high. Δ50 in this part of the parameter space increases by a factor of 3 for strong flood impacts, for weak flood impacts values are around 1.5. In contrast, premiums show both a positive relationship with *p* and *δ*, which is an outcome from Equation (13). At high values of *p* and *δ*, premiums are 20% of annual revenues.

## 4 Discussion

We have developed a mathematical insurance framework for quantifying the benefits and costs of cooperative indemnity insurance in small-holder aquaculture systems, using Myanmar as a case-study. Through ensemble simulations, we found that investing 5–20% of one's annual revenue into a risk pool's mutual fund can lead to large increases in the 5th percentile of income, or in other words a dramatic reduction in down-side risk. Interestingly, there is a non-monotonic relationship between this change in the downside of the income distributions and the probability of flooding *p*, and the fraction of loss covered *δ*. There are also benefits to individual fish farmers in terms of the change in median income experienced during a flood-year, which also increases, but shows a very different qualitative relationship with flood probability and fraction of loss covered.

**Figure 8:**
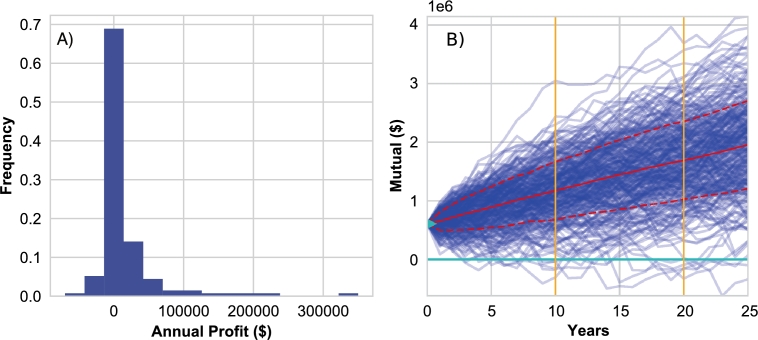
A) Empirical profit distributions for the Myanmar fish farm system, highlighting that there are several farms in 2016 with negative profits. B) Mutual fund trajectories from a Gamma risk pool, where premiums are modulated by a factor proportional to the risk that a given farm is exposed to (i.e. large farms pay disproportionally more relative to small farms).

While we have focused solely on income (revenue minus losses due to floods, accounting for premiums if paid), the data we have allow us to quantify the gross margin – profit – that the Myanmar fish farmers are likely to gain (see Figure 8A). Indeed, these data show that the margins are slim, with many fish-farmers accruing a loss in 2015, the year the MAAS interviews focused on (Belton et al., 2015). This highlights the importance of managing down-side risk in small-holder aquaculture with financial instruments like insurance. This is true for the fish farmers in Myanmar, but also for small-holder aquaculture communities around the world. For instance, in Bangladesh fish farmers are exposed to similar levels of flooding (Brouwer et al., 2007) and in Australia, it is drought that can have large negative impacts on fish farm production (Morrongiello et al., 2011). Although aquaculturists are exposed to great risk, except for a few recent pilot studies (Van Anrooy, 2006; Beach and Viator, 2008) there is little evidence that financial risk management tools are available for aquaculture enterprises around the world. This places a key limit on the (sustainable) growth of the industry (Secretan et al., 2007).

The work we have presented can be enhanced with several future extensions to the risk pool framework. For example, one of the main challenges a risk pool faces is ruin, which we dealt with by limiting the payouts claimants receive. However, there are several alternative solutions to this problem. For example the risk pool members could purchase reinsurance on their mutual fund. With such coverage, if ruin were to occur, the mutual fund would be filled back up to a given level. Another is to modulate the premium paid such that probability of ruin is diminished. One way of doing so is to add a term proportional to the standard deviation of the pay-out:

(16) $$\Pi_{i}=E[X_{i}]+\alpha \sigma (X_{i}).$$

Here $\sigma (X_{i})$ is the standard deviation of the payout $X_{i}$ and α>0 is a parameter that has to be set (Kaas et al., 2008; Mikosch, 2009). In our case we have

(17) $$\mathrm{Var}(X_{i})=\mathrm{Var}(\delta R_{i}I_{i}\xi_{i})=\delta^{2}R_{i}^{2}\mathrm{Var}(I_{i}\xi_{i})=:\delta^{2}R_{i}^{2}C^{2}.$$

Here, since neither the distribution of $I_{i}$ or that of $\xi_{i}$ depends on *i*, *C* is a constant independent of *i*. It follows that

(18) $$\sigma (X_{i})=\delta CR_{i}.$$

Introducing

(19) γ=αδC,

we see that we can write the modified premium as

(20) $$\Pi_{i}=\delta R_{i}pE[\xi_{i}]+\gamma R_{i}.$$

The second term on the right ($\gamma R_{i}$) effectively bends the mutual fund trajectories upwards (Figure 8B), away from the zero-line, and hence reducing the probability of ruin. Further, because premiums are increased in proportion to the revenue a farm makes, it is fair: i.e. larger farms pay more than smaller farms. We do not go any further with these “Gamma” risk pools, but this method can be used in conjunction with limiting mutual funds to positive values, and one fruitful future step is to determine the “optimal” value of *γ* such that ruin happens at a probability chosen by risk pool members. Further, by creating upward trajectories in the mutual fund, risk pool members could also choose an upper limit to the mutual fund, or else it will continue to grow, which will be of limited use to them. When a mutual cap is set, *γ* is then simply a parameter that governs how quickly the mutual fund reaches that limit. Ultimately, the choice of the mutual cap and *γ* will affect the ruin probability of the mutual fund, and hence the frequency by which risk pool members receive payouts smaller than their claims.

Two important choices for implementing a risk pool are how to model the probability of flooding and flood impacts. Here, we treated the probability of flooding as uniform across fish-farms, quantified using the data from one-year's worth of interviews. This choice reflects the large spatial scale of flooding in the Myanmar system (Belton et al., 2017) and the limitations set by the available data. However, in other systems, the spatial scale of flooding may be smaller than the spatial extent of the risk pool. In this case the probability of flooding would vary amongst risk pool members. Our mathematical framework can account for such a situation by appending a farm-index to the probability of flooding: $p_{i}$. Everything else follows as we have done so here, for example the calculation of premiums. Next, once a flood has occurred, we modeled the impact of the flood using a beta distribution, again after examining the empirical data. However, this need not be the case and other probabilistic models of flood impacts, for example a uniform distribution between a given minima and maxima, could be employed. Standard methods from distribution fitting can help identify the “best” flood impact model to use.

Perhaps the largest assumption in our simulation experiments was that every fish-farm in the Myanmar system formed one large risk pool. The size of the risk pool is important because, like all other forms of insurance, the larger the insurance pool, the more available capital there is to provide payouts and the more widely risk is spread, diminishing the likelihood of ruin. How then might risk pools initially form, when small pools are likely to be unattractive (i.e. with high premiums)? There are various answers to this question. Perhaps the initial set of fish-farmers have sufficiently strong social-ties and are willing to commit large fractions of their annual revenue to the mutual fund, knowing that as the pool grows over time, this cost will diminish. Another viable option would be for small risk pools to seek some form of subsidy, in the form of government support for example. This is common in other food-production systems. For example, fishermen are known to commonly form cooperatives that make decisions as a collective on where and how much to fish (McCay et al., 2014), and for dealing with risk (Sethi, 2010; Tilman et al., 2018). In many of these fisheries cooperatives, outside assistance is often required to get individuals to join the cooperative, for example through co-management with fisheries management agencies or indeed through subsidy (Pomeroy, 1995). Indeed, the viability of cooperative insurance schemes is a subject of much study in agriculture (Barnett and Mahul, 2007) and aquaculture (Nissapa et al., 2016; Xinhua et al., 2017), and a focus for technology innovation (e.g. https://www.worldcovr.com/), suggesting that while currently rare, cooperative risk pools in aquaculture will become more popular in the near future.

Risk pools offer a means to overcome some of the main challenges associated with traditional insurance. In particular, the main cost to insurance is typically in verifying claims, which is normally done via in-person monitoring (Miranda and Glauber, 1997). However, in the risk pool model claims could be verified by members of the pool. Further, making a false claim (e.g. pretending there has been a loss when there has not) is disincentivized because risk pools are formed around the often strong social-ties in fish-farming communities, and as a consequence the threat of social ostracism can deter bad behavior (Tavoni et al., 2012; Tilman et al., 2017). The problem of false claims is related to the broader challenge of moral hazard in insurance (Chambers, 1989). This is where fish farmers adopt more risky behaviors once insured, and it is currently unknown how moral hazard might manifest in a fish-farming risk pool. Like verifying claims, the problem of moral hazard could be solved, in part, by monitoring of farms/farmers by members of the cooperative. Another key challenge associated with insurance is adverse selection (Just et al., 1999), and risk pools are not immune to this problem: even though they rely on the social capital of fish-farming communities, members will still have an incentive to select against risky individuals (especially as in reality the probability of flooding *p* naturally varies amongst farmers), either by kicking-out risky members or by denying entry to risky farmers to the pool. Solution to the problem of adverse selection include offering a menu of insurance contracts, thus separating fish farmers with different risk profiles, including deductibles and using index triggers for payments.

In addition to forming an insurance risk pool, one very important step that the fish-farmers in Myanmar could make is to reduce the probability of flooding *p*. In the region of Myanmar that we have studied, the vast majority of ponds are located in an area that is already protected by flood defenses. These were constructed during the 1990s in order to make the area cultivable (Belton et al., 2017). This land is an area of low lying flood plain located between two of the major distributaries of the Ayeyarwady River. As such, during extreme flood events, existing flood control infrastructure is inadequate to prevent this area becoming inundated. Individual farms can (and do) invest in raising dykes. We envision that in addition to receiving direct payouts from the mutual fund to cover any losses, another useful scheme would be for fish-farming communities to invest part of their mutual fund in developing flood protection infrastructure (like dykes). In the long-run this would reduce the probability of flooding and/or limit losses due to floods, and ultimately reduce the premiums required of the members of the risk pool.

In small-holder agriculture, weather index or parametric insurance policies have grown in popularity due to efficiencies in overcoming challenges associated with false claims (Muller et al., 2017). This is when the probability of flooding, and the occurrence of a flooding event are calculated and identified from historical and real-time remotely sensed data respectively (Dalhaus and Finger, 2016). Weather index insurance is attractive in small-holder food production systems because it can be tailored on specific down-side events (Conradt et al., 2015) and it removes the problem of moral hazard (e.g. producers who stop working when insured) and false claims (e.g. claiming a loss has occurred when it has not). This approach could be useful for small-holder fish farmers too, and there are several initiatives dedicated to the analysis of remotely sensed data that is specific to quantifying risk in aquaculture production (Saitoh et al., 2011). The main challenge here is minimizing *basis risk*, which is essentially the error in the empirical relationship between an index derived from remotely sensed data, and the probability of loss (Barnett and Mahul, 2007). Basis risk in aquaculture can come from many sources: there will be error in the relationship between fish production and precipitation (at both extremes, from floods to droughts) and with temperature for example. However, basis risk can be effectively managed by many means, for example when indexes are used to trigger payouts for extreme weather events only (Conradt et al., 2015; Muller et al., 2017).

Possibly the most difficult aspect of weather-related food-production insurance are the stationary (e.g. oscillations) and non-stationary (i.e. changes in average conditions) aspect of local and regional environment. In Myanmar precipitation is known to exhibit long-term oscillations (Sen Roy and Sen Roy, 2011), and this could engender strategic entry and exiting behaviors amongst fish farmers. Further, we know that in the coming decades there are likely to be large changes in our climate and weather (Moss et al., 2010; Cochrane et al., 2009), with specific implications for aquaculture (Klinger et al., 2017). This means that, in addition to the various choices over the heterogeneity in flood probabilities and how to model flood impacts, these factors are also changing in time. There are several methods for dealing with non-stationarity, caused for example by climate change (Kapphan et al., 2012), the simplest being a moving window assessment of the risk pool parameters. However, in the limit of extreme climate change and long-time scales (i.e. decades), there are situations where a risk pool that is “viable” now, in terms of its premiums that fish-farmers are willing to pay and the payouts they receive, will cease to be in 10–20 years. It is an open question as to how to deal with these situations, but it will likely involve using additional financial tools to help risk pool members transition to new sources of income and food.

The modeling framework we have developed, while specific to small-holder aquaculture in Myanmar, could be applied to any other system comprised of individually-owned food production facilities that are affected by adverse weather. These could be crop or cattle-farms, fishermen, vineyards, or even ski resorts. This is because the risk pool framework is built from simple advances to general insurance mathematics. Indeed, the motivation for this work came from Protection and Indemnity Clubs, which are some of the oldest forms of cooperative insurance (Bennett, 2001). More broadly, there appear to be numerous opportunities to borrow financial tools (like cooperative insurance) from other fields and economic sectors and apply them to the aquaculture industry. Financial risk management tools, like futures and forward contracts and other forms of insurance, are widely used in agriculture and other food production sectors (Moschini and Hennessy, 2001; Flaten et al., 2011). But they are relatively little used in aquaculture (Secretan et al., 2007), which is currently the fastest growing food-production sector on the planet (Klinger and Naylor, 2012). We are at a critical point in the development of the aquaculture industry because these financial tools, if designed poorly, can incentivize environmentally harmful behavior (Muller et al., 2017). A key next-step in this work, and in the progression of the aquaculture industry more generally, is to identify how new financial tools can be designed for the opposite effect. In doing so, the use of financial tools like cooperative insurance, could help steer the aquaculture industry towards sustained growth and multiple wins, including enhanced financial risk management and long-term profitability for aquaculture enterprises and reduced environmental intensity of the production through adoption of best management practices.

## 5 Conclusions

Aquaculture is a rapidly growing industry, providing food and income to many millions of people around the world. However, it is an immature sector relative to other food producing sectors, especially in terms of the risk management tools available. Here, we have developed a cooperative indemnity insurance scheme that is tailored to a fish-farming community in Myanmar. The insurance scheme revolves around members of a community pooling funds to protect against losses incurred by floods, which are common in the region. The scheme greatly reduces the downside risk of fish-farmers, and ultimately provides resilience to the community through smoothed income. These forms of cooperatively managed and (potentially) self-organized insurance are a promising route by which the aquaculture industry can maintain a positive trajectory in terms of its growth, while achieving economic gains through reduced risk for the producer, and environmental wins through incentivizing best management practices.

## Declarations

### Author contribution statement

James R. Watson, Fredrik Armerin, Dane H. Klinger, Ben Belton: Conceived and designed the experiments; Performed the experiments; Analyzed and interpreted the data; Contributed reagents, materials, analysis tools or data; Wrote the paper.

### Funding statement

This work was supported by the NSF Dynamics of Coupled Natural-Human Systems project GEO-1211972, the USAID Food Security Policy Project AID-482-LA-14-00003, the Livelihoods and the Food Security Trust Fund Agrifood Value Chain Development in Myanmar project: Implications for Livelihoods of the Rural Poor and The Global Economic Dynamics and the Biosphere Programme at the Royal Swedish Academy of Sciences.

### Competing interest statement

The authors declare no conflict of interest.

### Additional information

No additional information is available for this paper.
